# Comparative Evaluation of Dexmedetomidine and Dexamethasone as Adjuvants in Supraclavicular Brachial Plexus Block

**DOI:** 10.7759/cureus.38775

**Published:** 2023-05-09

**Authors:** Nagaraju A, Lingaraj Sahu, Sudeepa Das, Manoja Muni

**Affiliations:** 1 Anesthesiology, Employee’s State Insurance Corporation (ESIC) Medical College and Hospital, Hyderabad, IND; 2 Anesthesia, Kalinga Institute of Medical Sciences, Bhubaneswar, IND; 3 Anatomy, Kalinga Institute of Medical Sciences, Bhubaneswar, IND

**Keywords:** upper limb surgeries., supraclavicular brachial plexus block, ultrasound guidance, bupivacaine, dexamethasone, dexmedetomidine

## Abstract

Introduction

Currently, peripheral nerve block has shown immense potential with effective patient satisfaction. In the event of upper limb surgeries, the supraclavicular brachial plexus approach under ultrasound guidance renders quick and dense anesthesia. In addition, the clinical utility of adjuvants with local anesthetics elicits a good quality of nerve block with improved duration and inset. So the aim of the present study was to compare the block characteristics of dexmedetomidine and dexamethasone during supraclavicular brachial plexus block in patients undergoing upper limb surgeries.

Materials and methods

The present study was conducted on 100 patients aged 20-60 years with the American Society of Anesthesiologists (ASA)-I and ASA-II classification who were scheduled for upper limb surgeries. These patients were divided equally into two groups, namely group D (who received 20mL of 0.5% bupivacaine + 50 mcg (0.5mL) of dexmedetomidine +1.5mL normal saline) and group X (who received 20mL of 0.5% bupivacaine +8mg of dexamethasone), ensuring a total volume of 22mL administered to both groups. The time of onset and duration of the sensory and motor blocks, as well as the quality of intraoperative analgesia, were assessed.

Results

The addition of dexmedetomidine (50mcg) and dexamethasone (8mg) to 0.5% bupivacaine ensured a faster onset and prolonged duration of the sensory and motor blocks. Additionally, dexmedetomidine resulted in more prolonged postoperative analgesia, a lower mean visual analog scale score in the first 24 hours, and lesser opioid consumption in 24 hours than dexamethasone.

Conclusion

Dexmedetomidine is superior to dexamethasone as an adjuvant to bupivacaine during supraclavicular brachial plexus block in patients undergoing upper limb surgeries.

## Introduction

A brachial plexus block is an effective alternative to general anesthesia for upper limb surgeries. It also ensures a low-stress response and minimal anesthetic use, which thus provides better intraoperative analgesia and extended postoperative pain relief. A wide range of brachial plexus blockade methods is used, such as interscalene, supraclavicular, infraclavicular, and axillary. The upper limb usually consists of a rich nerve supply; supraclavicular brachial plexus block (SCBP) ensures dense anesthesia with rapid onset of action [1]. To increase the block outcome, a wide range of adjuvants have been added with local anesthetics (LAs). Adjuvants such as midazolam, clonidine, hyaluronidase, neostigmine, bicarbonate, and dexamethasone have been used clinically in combination with LAs for the augmentation of peripheral nerve block and also for increasing postoperative analgesia [2-7]. The use of adjuvants minimizes the toxic effects of LAs by reducing the total dose. An ultrasound-guided brachial plexus block provides quick and dense anesthesia due to accurate needle placement and also reduces the dose of the LAs with a good safety profile. Dexmedetomidine exhibits a high affinity for alpha 2 receptors, and when supplemented with bupivacaine, it results in a rapid onset of action with prolonged block duration and enhanced postoperative analgesia [5,8-11]. Likewise, dexamethasone is a highly selective long-acting glucocorticoid with a higher potency and prolonged analgesia up to 48 hours [12]. The action of dexamethasone is due to its vasoconstriction effects, which decrease the local anesthetic absorption. In addition, it inhibits the potassium channels on its nociceptive C-fiber and blocks the release of various inflammatory mediators. We hypothesized that dexmedetomidine would be as effective as dexamethasone as an additive in the supraclavicular nerve block. In this randomized, double-blind prospective study, the primary objective was to compare the block characteristics of dexmedetomidine and dexamethasone as adjuvants to bupivacaine under ultrasound guidance for supraclavicular brachial plexus block in patients undergoing upper limb surgeries. The secondary objective was to compare the onset and duration of sensory and motor blocks and postoperative analgesia in the first 24 hours during supraclavicular brachial plexus block in patients undergoing upper limb surgeries.

## Materials and methods

Materials and methods

The present randomized double-blind prospective study was conducted in 100 patients aged 20-60 years with the American Society of Anesthesiologists (ASA)-I and ASA-II classifications scheduled for upper limb surgeries in the Department of Anesthesia, KIMS. Institutional ethics clearance was obtained before the study [KIMS/KIIT/IEC/152/2018; Clinical Trial Registration of India vide no.: CTRI/2019/07/020417]. Written informed consent was obtained from all subjects, the legal surrogate, the parents or legal guardians for minor subjects, or the requirement for written informed consent was waived by the IRB. 

Sample size calculation

A pilot study was carried out before the actual study. We included nine patients in each group; the mean (±SD) VAS score in group D, the dexmedetomidine-treated group, was 1.8 ±.2 and 3.5 ±1.5 in group X, the dexamethasone-treated group. According to this pilot study, with a significance level of 0.05 and a potency of 0.8, we included 50 cases in each group to determine a clinically relevant reduction in pain level.

Inclusion criteria

Patients with ASA I and II scheduled for upper limb surgeries and patients aged between 20 and 60 years were included in the study.

Exclusion criteria

Patients exhibiting reluctance to participate in the study (sepsis at the injection site, history of coagulopathies, and history of hypersensitivity to bupivacaine, dexamethasone, and dexmedetomidine) were excluded from the study.

Study design

The 100 patients undergoing lower limb surgeries were randomly divided into two groups (n=50) as follows: 

Group D (n=50): Received 20mL of 0.5% bupivacaine + 50mcg (0.5mL) of dexmedetomidine +1.5mL normal saline, 

Group X (n=50) Received 20mL of 0.5% bupivacaine +8mg of dexamethasone, ensuring a total volume of 22mL.

The drug solutions were prepared by an anesthesiologist who was not involved in the study. The anesthesiologist performing the block and observing the patients was blind to the treatment groups. All the data were compiled by an anesthesiologist who was also blind to the group allocation. A pre-anesthesia assessment was performed the day before surgery. The patients were informed of the visual analog scale (VAS) for postoperative pain assessment. On this scale, 0 indicates no pain, whereas 10 indicates severe pain.

All baseline vital parameters were recorded before surgery. Ringer lactate solution was administered intravenously by using an 18G cannula. The ultrasound-guided supraclavicular block was performed with the patient in a semi-sitting position, and the head rotated to the opposite side of the side to be blocked. Before the procedure, the site was disinfected, and the ultrasound probe was kept in the coronal oblique plane in the supraclavicular fossa. The hypoechoic pulsating subclavian artery was recognized above the hyperechoic first rib and confirmed by color Doppler. The probe was positioned until both the first rib and pleura were seen simultaneously while keeping the artery in view. The supraclavicular brachial plexus was identified as the structure mimicking a bunch of grapes. The proposed puncture site was infiltrated with 1 mL of 2% lidocaine. Then, the needle was inserted in a lateral to the medial plane until the brachial plexus was reached. A “palpable pop” confirmed the insertion of the needle into the sheath. After confirming negative aspiration for blood, 1-1.5mL of the prepared drug was injected to confirm the needle placement. After confirming the proper dissemination of the drug around the brachial plexus, further progression of the needle by 1-2 mm was performed to achieve ample spread of the prepared drug. After injecting the prepared drug, the block was tested for both sensory blockage and motor blockage by using the spirit swab method and the Bromage score, respectively, with 0 indicating no block and 3 indicating total block. If a motor block of Bromage 3 was not achieved within 30 min, the block was considered to have failed, and those patients were ruled out of the study. The maximum allowable doses in 24 hours for paracetamol and tramadol were 4 g and 300 mg, respectively. If the patient had persistent VAS ≥6 after receiving paracetamol and tramadol, morphine 0.1 mg/kg IV was given as a rescue analgesic.

The data were compiled using MS Excel (Redmond, USA), and the statistical analysis was performed using IBM Corp. Released 2011. IBM SPSS Statistics for Windows, Version 20.0. Armonk, NY: IBM Corp. A p-value < 0.05 was considered statistically significant.

## Results

There was no significant difference in, age, gender, ASA physical status, body mass index (BMI), or duration of surgery between groups D and X, as shown in Table 1.

**Table 1:** Comparison of demographics and clinical characteristics between the groups D: Dexmedetomidine; X: Dexamethasone; BMI: Body mass index; ASA: American Society of Anesthesiologists; SD: Standard deviation

Demographics and Clinical characteristic		Group D	Group X	p-value
Age (years)	Mean ± SD	36.08±13.360	35.58±11.585	0.842
Sex	Men	33 (66%)	34(68%)	1
	Women	17(34%)	16(32%)	
ASA status	1	40 (80%)	40(80%)	1
	2	10 (20%)	10(20%)	
BMI(kg/m^2^)	Mean ± SD	27.024±2.8700	26.978±3.2062	0.94
Duration of surgery(min)	Mean ± SD	100.46±27.542	101.70±23.509	0.809

The onset and duration of sensory and motor block among the groups are shown in Table 2. In group D, the onset of sensory and motor blocks was found to be significantly lower than in group X (8.34±1.49 minutes vs. 9.06±1.44 minutes, p = 0.016) and (12.02 ± 1.45 minutes vs. 13.54 ± 1.38, p < 0.001), respectively. The duration of sensory and motor block was significantly higher in Group D as compared to Group X 866.98 ± 125.43 minutes vs. 834.72 ±125.43 ; p<0.001) and (747.64 ± 104.50 minutes vs. 726.44 ± 100.58 minutes; p<0.001).

**Table 2:** Comparison of the onset and duration of sensory block between the groups D: Dexmedetomidine; X: Dexamethasone. * denotes significant p <0.05.

Block parameters	Group D (Mean±SD)	Group X (Mean±SD)	p-value
Onset of sensory block (min)	8.34±1.493	9.06±1.449	0.016*
Onset of motor block (min)	12.02±1.450	13.54±1.388	<0.001*
Duration of sensory block (min)	866.98 ± 125.432	747.64 ± 104.502	< 0.001*
Duration of motor block (min)	834.72 ± 125.432	726.44 ± 100.585	< 0.001*

The analgesic requirement and VAS score in the first 24 hours among the groups are shown in Table 3. The time needed for the first analgesic requirement was significantly higher in Group D than in Group X (935.38 ± 129.01 vs. 810.66 ± 107.01 minutes p < 0.001) and found to be significant. The number of analgesics required in Group D was significantly lower than in Group X (1.56 ± 0.50 vs. 1.98 ± 0.58, p =0.001). The mean VAS score was significantly lower in Group D as compared to Group X in the first 24 hours (2.98 ± 0.80 vs. 3.427 ± 0.7409, p = 0.00). 

**Table 3:** Comparison of the visual analogue score and analgesic requirements between the groups VAS: visual analogue scale, *:statistically significant, p<0.05

Analgesia efficacy parameters	Group D (Mean±SD)	Group X (Mean±SD)	p-value
First analgesic requirement (min)	935.38 ± 129.011	810.66 ± 107.015	< 0.001*
Number of analgesics given in the first 24 h	1.56 ± 0.501	1.98 ± 0.589	< 0.001*
Mean VAS score during first 24 h	2.980 ± 0.8036	3.427 ± 0.7409	0.005*
Total tramadol requirement in the first 24h (mg)	161.60 ± 51.085	197.60 ± 50.611	0.001*
Paracetamol requirement (gm)	0.14 ± 0.351	0.26 ± 0.443	0.136

## Discussion

Dexmedetomidine exhibits a high affinity for alpha-2 receptors, and its optimal dose is dependent on its sedative effect, analgesia, and hemodynamic properties. Previous clinical studies show that dexmedetomidine in the dose range of 30-100 µg is used for brachial plexus blocks (BPBs) [8,13]. Due to its peripheral analgesic action, dexmedetomidine might decrease the onset time and duration of the motor and sensory blocks while increasing analgesia [14]. Dexamethasone, when added to plain ropivacaine 0.5%, can be used for supraclavicular brachial plexus block and elicits a decreased sensory and motor block onset time and also increases the duration of sensory and motor block. Further, 8 mg dexamethasone along with 0.5% levobupivacaine in supraclavicular brachial plexus block leads to reduced demand for rescue analgesics with a brisk onset of block and extended sensory and motor block duration. So the present study compared the effects of dexmedetomidine (50mcg) and dexamethasone (8mg) as adjuvants to 0.5% bupivacaine in supraclavicular brachial plexus block.

In the present study, the dexmedetomidine group exhibited a faster onset and more prolonged duration of the sensory and motor block, longer postoperative analgesia, a lower mean VAS score in the first 24 h, and lesser opioid consumption in the first 24 h than the dexamethasone group.

Similar to our findings, previous two randomized double-blinded trials by Bisui et al. and Kaur et al. and a meta-analysis done by Abdallah and Brull showed that the addition of dexmedetomidine to local anesthetic in the brachial plexus block was reported to shorten the onset time of the sensory block [15-17]. Adinarayanan et al. used a control group with the dexmedetomidine and dexamethasone groups [18], whereas Singh et al. compared dexmedetomidine and dexamethasone in the brachial plexus block under ultrasound guidance [19]. The outcome of these studies shows that the dexmedetomidine group displayed a significantly faster onset of the motor block than the dexamethasone group. The results are consistent with the present study findings, which indicate that the onset of the motor block was significantly faster with dexmedetomidine than with dexamethasone.

In our study, the duration of the sensory and motor blocks was significantly longer with dexmedetomidine as compared to dexamethasone. Our results are in corroboration of the studies conducted by Adinarayanan et al., Gao et al., Albrecht et al., and Elyazed et al., respectively [18,20-22]. Previous studies done by Hamada et al. and Yadav et al. reported that the time needed for the first analgesic requirement is significantly longer in the dexmedetomidine group as compared to the dexamethasone group, which is in line with the present study [23,12]. In our study, the mean VAS score during the first 24 h was significantly lower in the dexmedetomidine group as compared to the dexamethasone group, which is in line with previous reports [24]. In addition, the total tramadol dose required in the first 24 h was significantly lower in the dexmedetomidine group as compared to the dexamethasone group, and in the Niranjan et al. study, similar findings were observed [24]. Meanwhile, in our study, the difference in the mean paracetamol requirement between the dexmedetomidine group and dexamethasone was found to be non-significant. 

Limitations

The present study has certain limitations. The equipotent dose of dexmedetomidine and dexamethasone could not be calculated. Additionally, the evenness of the preoperative pain threshold could not be ensured among all the patients, and analgesics were administered as per the patients’ sensitivity to the pain (VAS ≥ 3).

## Conclusions

We conclude that the addition of dexmedetomidine or dexamethasone as an adjuvant to bupivacaine effectively decreased the time of onset for the sensory and motor blockade, the mean VAS score, and opioid consumption. Additionally, it increased the duration of the motor block, sensory block, and postoperative analgesic period. Meanwhile, dexmedetomidine is superior when compared to dexamethasone in terms of quality of block.
